# Letter to the Editor Josefsson et al. ‘Reduced periprosthetic fracture rate for a cemented anatomical versus a tapered polished stem in hip arthroplasty: A 6‐year follow‐up of a prospective observational cohort study’

**DOI:** 10.1002/jeo2.70412

**Published:** 2025-09-09

**Authors:** Peter Wahl, Emanuel Gautier

**Affiliations:** ^1^ Endo‐Team Birshof Hospital Muenchenstein Switzerland; ^2^ Department of Biomedical Engineering University of Basel Allschwil Switzerland; ^3^ ARTORG Centre for Biomedical Engineering Research, Faculty of Medicine University of Bern Bern Switzerland; ^4^ Department of Orthopaedic Surgery HFR Fribourg – Cantonal Hospital Fribourg Switzerland

To the Editor,

We read with great interest the recent publication in the *Journal of Experimental Orthopaedics* by Josefsson et al., entitled ‘Reduced periprosthetic fracture rate for a cemented anatomical versus a tapered polished stem in hip arthroplasty: A 6‐year follow‐up of a prospective observational cohort study’ [4]. As long‐standing proponents of the view that classifying fixation in total hip arthroplasty merely as ‘cemented’ or ‘uncemented’ is overly simplistic, not doing justice to essential outcome determinants, we were pleased to see the authors address differences between cemented stems using either a force‐closed (or taper‐slip) or a shape‐closed concept. These distinctions are often underappreciated, despite their clinical relevance. Notably, variation in revision rates between fixation principles have been observed, particularly higher and progressively increasing revision risks associated with mixed concepts such as the French paradox technique [7]. Among the pure concepts, shape‐closed designs perform much better than force‐closed designs regarding the risk of periprosthetic fractures (PPF) of the proximal femur, as corroborated by both the present study [4] and a recently published systematic review of the literature with meta‐analysis [6].

**Figure 1:**
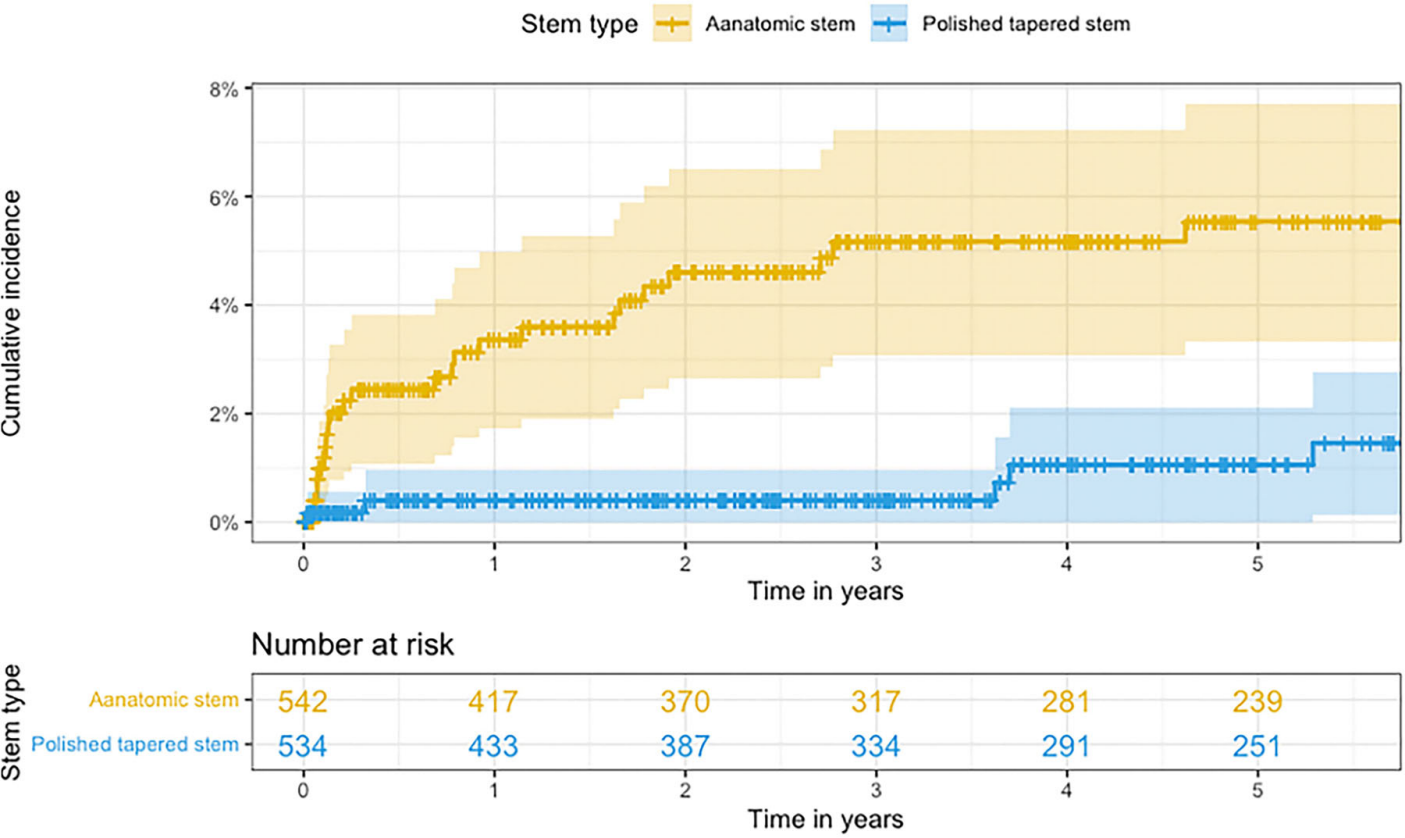
Figure 1 from the original publication by Josefsson et al. The legend does not correspond to the description and should be inverted.

However, we would like to draw attention to a likely error in Figure 1, where the legend appears to have been inverted. Given the importance of this figure in conveying the study's key findings, we suggest issuing a corrigendum: the orange curve should correspond to polished tapered stems, and the blue curve to anatomic stems.

**Figure 2:**
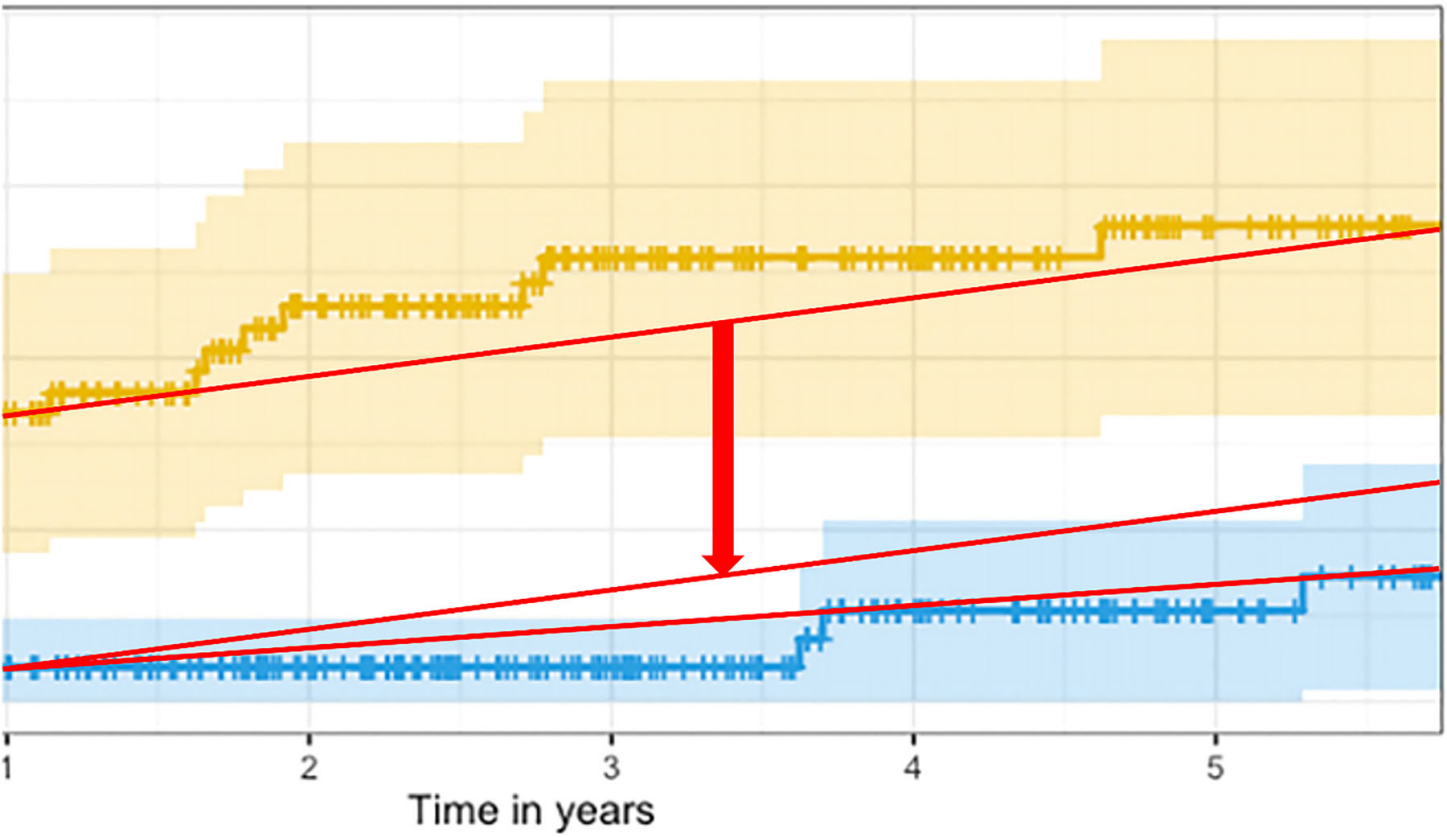
Magnification of Figure 1 from the original publication by Josefsson et al. Note the difference in increase over time of the revision rate.

Beyond this, we believe the results merit a more nuanced discussion, particularly in light of potential confounding factors not addressed in the analysis. The temporal pattern of occurrence of PPF after THA in the force‐closed stem group appears atypical, resembling more closely the early postoperative profile of uncemented stems [1]. This may reflect intraoperative issues rather than inherent shortcomings of the cementation concept. The distribution of fracture types (illustrated in Figure 2 of the original publication) might similarly indicate surgical technique issues, such as overly aggressive broaching. Additional design features of the collarless polished taper (CPT) stem (Zimmer Biomet, Zug, Switzerland)—such as its length, its shoulder profile requiring sufficient lateral broaching, and the integrated cement mantle of 2 mm incorporated into the broaches of Exeter designs—may have contributed to technical difficulties, particularly in patients with narrow medullary canals. Furthermore, the higher dislocation rates in the force‐closed group might reinforce concerns regarding a suboptimal technique. This interpretation is further supported by the uncommonly high rate of periprosthetic joint infection (PJI) in this study, as the global rate of PJI was 2.6%, and particularly high in the force‐closed group with 3.6%, compared to a commonly accepted 1% rate for THA [2, 3]. Excluding early fractures—those most plausibly linked to intraoperative issues ‐ the difference between the revision rates narrows and becomes no more significant, as the confidence intervals largely overlap, even if the increase rate seems higher in the force‐closed group (Figure 2).

Finally, it is worth noting that the CPT stem the authors used in the force‐closed group is well‐known to have higher revision rates than the Exeter stem it evolved from. This may well be attributable to the use of a cobalt‐chromium alloy for this stem rather than stainless steel, which has been associated with a hazard ratio of 6.7 regarding revision risks [5].

While we commend the authors for their valuable contribution, we would caution against generalising the findings to all force‐closed stems. The observed outcomes may be specific to the CPT stem and potentially even be specific to local surgical factors.
